# Diagnosis of Focal Nodular Hyperplasia (FNH) after Liver Transplantation

**DOI:** 10.1155/2020/8824099

**Published:** 2020-10-02

**Authors:** Christina S. Gainey, Suzanne L. Palmer, Edward Mena, Navpreet Kaur, Yuna Gong, Jeffrey A. Kahn

**Affiliations:** ^1^Department of Internal Medicine, LAC+USC Medical Center, Keck School of Medicine of USC, USA; ^2^Department of Radiology, Keck School of Medicine of USC, USA; ^3^California Liver Research Institute, Pasadena, CA, USA; ^4^Department of Surgery, University of Southern California, Los Angeles, CA, USA; ^5^Department of Clinical Pathology, Keck School of Medicine of USC, USA; ^6^Division of Gastrointestinal and Liver Diseases, Keck School of Medicine of USC, USA; ^7^Liver Transplant Program, Keck School of Medicine of USC, USA

## Abstract

Following liver transplantation (LT), recipients can develop benign and malignant hepatic masses just like any other patient. Patients transplanted for hepatocellular carcinoma (HCC) undergo surveillance imaging, and any new mass seen on imaging must be carefully evaluated to rule out recurrent cancer. Focal nodular hyperplasia (FNH) is a benign tumor of the liver that most often occurs in women and is rarely symptomatic. It is important to distinguish FNH from more serious etiologies, such as recurrent HCC and other malignancies, since the treatments differ greatly. To date, there have been very few reports of FNH occurring in a liver allograft. We present a case of a patient with a history of a carcinoid tumor who underwent LT for HCC. Several years posttransplant, the patient was found to have a liver mass with classic features of HCC on imaging. The liver biopsy revealed the unexpected diagnosis of FNH. This finding avoided unnecessary treatment for HCC, which is associated with morbidity, especially in the posttransplant setting. We present our diagnostic approach, discuss the clinicopathologic and imaging findings of FNH, and review the literature on FNH in the posttransplant setting.

## 1. Introduction

Focal nodular hyperplasia (FNH) is a benign liver lesion consisting of hyperplastic hepatocytes whose proliferation is thought to be driven by alterations in vascular perfusion. FNH has been well-characterized in native livers, but only a few cases describe its development in a liver allograft. We present a rare case of a biopsy-proven FNH-like lesion in a posttransplant liver that showed classic findings of HCC on imaging. We discuss the clinicopathologic and imaging features of FNH to help guide the diagnosis of hepatic nodules in liver allografts and prevent unnecessary treatment. We propose that FNH and FNH-like lesions should be considered in the differential diagnosis of hepatic nodules in posttransplant livers, especially in patients with a history of alterations in hepatic vascular perfusion. The clinical implications of accurately diagnosing the lesion to guide management cannot be overstated.

## 2. Case Presentation

A 64-year-old male with a history of cirrhosis due to nonalcoholic fatty liver disease, complicated by biopsy-proven multicentric HCC. Past medical history is significant for a remote history of pulmonary sarcoidosis as well as significant hypercoagulability. In addition to portal and splenic vein thromboses, the patient suffered numerous recurrent small bowel infarctions and bowel resections in both the pre- and postliver transplant settings. Hematologic work-up revealed an acquired protein C deficiency, likely secondary to his liver disease. An incidental finding on the pathology of resected bowel revealed a carcinoid tumor of the small bowel. Nuclear medicine octreotide scan revealed that the patient was free of active disease.

The patient underwent four sessions of transarterial chemoembolization (TACE) before undergoing deceased donor liver transplantation (total hepatectomy with caval preservation and duct-to-duct anastomosis). Pathology of the explant revealed multifocal, well-differentiated HCC, with no evidence of lymphovascular invasion. There were 4 tumor foci with a total viable tumor burden of 2.72 cm; the largest tumor measured 2.1 cm.

The patient's posttransplant course was complicated by recurrent portal vein thrombosis and was started on low molecular weight heparin. An episode of mild acute cellular rejection responded well to treatment with steroids. For several years following the transplant, the patient continued to obtain regular surveillance imaging and alpha-fetoprotein levels, with no evidence of abnormalities.

**Figure 1:**
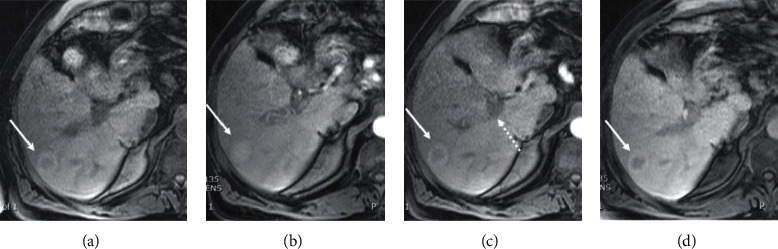
MRI with Eovist® (gadoxetate disodium contrast) shows the persistent portal vein thrombosis (dotted arrrow) and the segment 7 lesion (solid arrow). The lesion measures 2.6 cm and is faintly hypointense with hyperintense rim on precontrast T1W image (a), enhances centrally on arterial phase (b), central washout with a persistent hyperintense rim on portal venous phase (c), and no Eovist® uptake centrally with a persistent hyperintense rim on hepatocyte phase. This appearance is suspicious for HCC.

**Figure 2:**
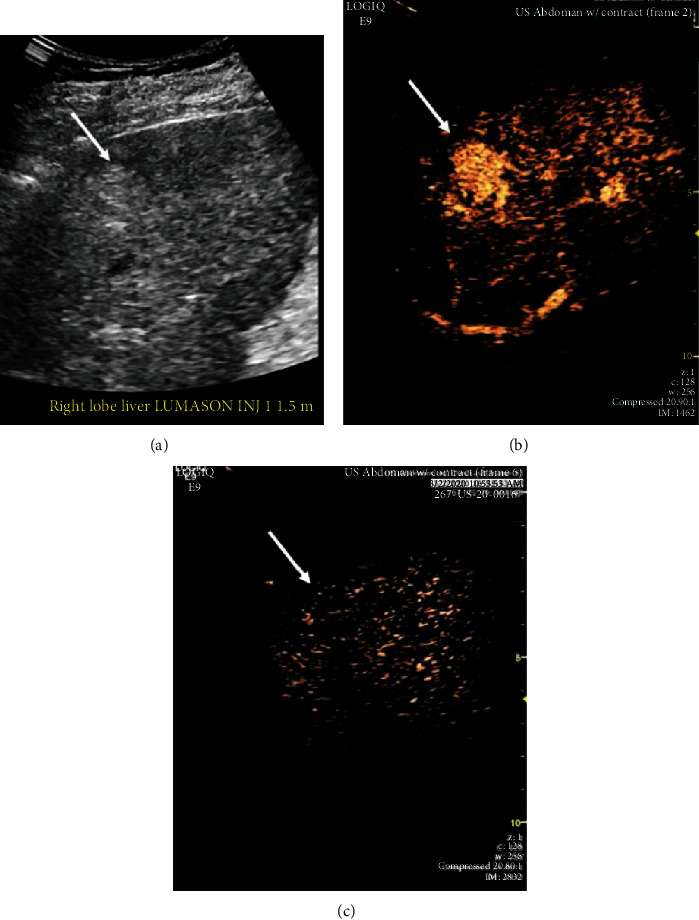
Contrast-enhanced ultrasound (US) demonstrates a 2.5 cm mass in segment 7 (arrow) that is echogenic on grayscale (a) and has early enhancement relative to the liver parenchyma (b). At 5 minutes postcontrast injection, there is a very subtle washout (c).

**Figure 3:**
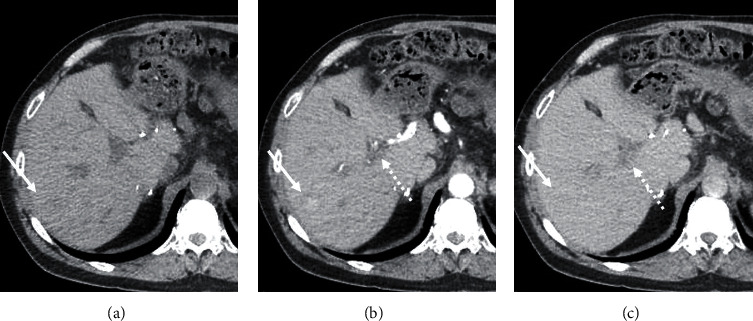
Retrospective review of a contrast-enhanced CT of the liver performed 8 months prior to contrast-enhanced MRI demonstrates portal vein thrombosis (dotted arrow) and a 2.3-cm lesion in segment 7 (arrow) that is faintly hypodense on precontrast (a), enhances centrally with a hypoenhancing rim on arterial phase (b), and subtly hypodense with a subtle rim of hyperenhancement on 3 min delay phase (c). In a patient with chronic liver disease, this appearance would be suspicious for HCC.

Five years following LT, the patient acutely presented with abdominal pain, nausea, vomiting, and diarrhea. Laboratory values included alkaline phosphatase 126 U/L, AST 42 U/L, ALT 80 U/L, total bilirubin 0.7 mg/dL, and alpha-fetoprotein 1.6 ng/mL. MRI with Eovist® (gadoxetate disodium contrast) showed a 26-mm hepatic lesion with arterial hyperenhancement and associated washout, consistent with hepatocellular carcinoma (OPTN 5B) (Figure 1). Contrast-enhanced ultrasound also showed distinct arterial enhancement and delayed subtle washout—again consistent with hepatocellular carcinoma (Figure 2). We later discovered that a CT scan had been done at an outside institution eight months prior to the MRI. The retrospective review of the contrast-enhanced CT scan revealed a 23 mm intrahepatic lesion with arterial enhancement and very subtle washout, suspicious for HCC (Figure 3). Given the conflicting clinical and imaging findings, we proceeded to percutaneous liver biopsy for definitive evaluation of the mass.

### 2.1. Microscopic Diagnosis of FNH and Differential Diagnosis

**Figure 4:**
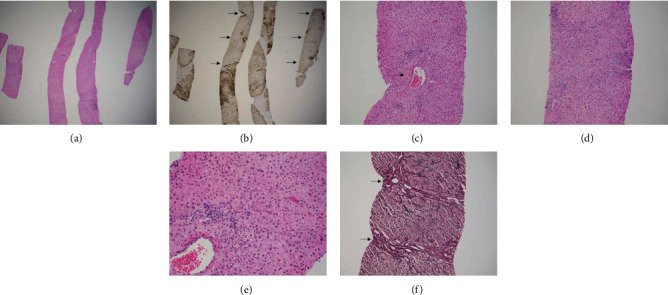
(a) The boundary between normal liver tissue and the lesional tissue is difficult to appreciate on H&E stain on low power (H&E stain, 20x magnification). (b) However, immunohistochemical stain for glutamine synthetase highlights the lesional area with patchy brown staining. Nodular architectural typical for FNH is also more evident. Adjacent normal liver tissue (arrows) shows the characteristic perivenular staining only (glutamine synthetase immunohistochemical stain, 20x magnification). (c) Large abnormal muscular arteries (arrow) without associated bile ducts of similar size are seen, a characteristic feature of FNH (H&E stain, 100x magnification). (d) Large fibrous tracts with ductular proliferation, similar to that seen in cirrhotic livers, is seen within the lesion (H&E stain, 100x magnification). (e) On high power, the hepatocytes within the lesion appear similar in appearance to the background nonlesional hepatocytes. No significant cell plate architectural abnormality or cytologic atypia diagnostic of hepatocellular carcinoma is identified (H&E stain, 200x magnification). (f) Reticulin stain highlights fibrous tracts within the lesion (arrows). Also, significant thickening of the hepatocellular plates, a feature of hepatocellular carcinoma, is absent (reticulin stain, 100x magnification).

Microscopic examination of the liver biopsy (Figure 4) revealed a lesion composed of cytologically normal-appearing hepatocytes arranged in incomplete nodules (panels (a) and (e)). These incomplete nodules are separated by thick fibrous tracts with ductular proliferation, similar to those seen in cirrhotic livers (panel (d)). Normal portal tracts are notably absent. Instead, large muscular arteries without associated bile ducts of similar size (panel (c)) are seen within fibrous tracts. In FNH, immunohistochemical (IHC) stain for glutamine synthetase will often show a distinctive “map-like” patchy staining of the hepatocytes, but this feature is difficult to appreciate in thin needle core biopsies (panel (b)). Reticulin stain (highlighting fibrosis) also typically demonstrates an intact framework in FNH (panel (f)). All of these morphologic features (e.g., bland cytologic features, presence of nodules, fibrous tracts with ductular proliferation and abnormal vessels) and staining patterns for glutamine synthetase and reticulin are most consistent with the diagnosis of FNH.

In contrast, the diagnosis of HCC requires cell plate architectural abnormalities and at least some degree of cytologic atypia. The cell plates in HCC are often arranged in trabeculae, pseudoglands, or solid nests, and the tumor cells typically show features of cytologic atypia (e.g., high nucleus-to-cytoplasm ratio, nuclear pleomorphism, and prominent nucleoli). None of these abnormal growth patterns or significant cytologic atypia were observed in our case. Furthermore, HCCs will show loss of normal reticulin framework and characteristic IHC staining pattern (diffuse positivity with glutamine synthetase and positivity with glypican-3 [1]). Our case did not show diffuse staining by glutamine synthetase (panel (b)) and was negative for glypican-3 (not pictured).

Given the patient's history of a small bowel carcinoid tumor, we were careful to consider a neuroendocrine tumor in our differential. Carcinoid tumors (well-differentiated neuroendocrine tumors) can have some morphologic overlap with hepatocellular lesions. Similar to HCCs, carcinoid tumors can show abundant eosinophilic cytoplasm and similar architectural patterns. In contrast to hepatocellular lesions, however, carcinoid tumors have a very characteristic “salt and pepper” chromatin pattern without conspicuous nucleoli. If morphologic features are insufficient for the distinction between the two entities, IHC stains for neuroendocrine markers (e.g., synaptophysin and chromogranin) and hepatocellular markers (e.g., Arginase-1 and HepPar-1) will typically show the opposite staining patterns in hepatocellular lesions and carcinoid tumors. In our case, morphologic features were sufficient to exclude neuroendocrine differentiation, and these stains were not performed.

## 3. Discussion

### 3.1. Background

Focal nodular hyperplasia (FNH) is the second most common hepatic lesion, with the most common being hepatic hemangioma. The reported prevalence of FNH in the United States is estimated to be around 0.03%-3% based on autopsy studies [2] and 0.2% based on US findings [3]. FNH is primarily diagnosed in females 40-50 years old, with males making up fewer than 15% of the cases. Interestingly, FNH lesions in males are generally noted to be smaller with more atypical pathology [4]. Most cases of FNH are asymptomatic and stable over time; management is conservative [5].

### 3.2. Imaging Characteristics of FNH and Potential Pitfalls

FNH can be difficult to detect on imaging studies. When seen, it is usually due to mass effect and not differences in echogenicity (ultrasound), density (CT), or intensity (MRI). Classic appearance on contrast-enhanced multiphase CT and MRI studies is arterial hyperenhancement that approaches the density or intensity of the liver parenchyma in both the portal venous and delayed phases. When using a biliary agent, such as gadoxetate disodium, all but the central scar will demonstrate contrast uptake on the hepatocyte phase of the MRI. On the contrast-enhanced US, enhancement of the central scar appears first, with progressive centrifugal enhancement and a sustained mild enhancement on the portal venous phase. A large feeding vessel may be seen on the arterial phase [6].

Given the paucity of literature describing FNH-like lesions in posttransplant livers, many would not consider FNH as a possible diagnosis. FNH and FNH-like nodules can be difficult to distinguish from HCC on imaging alone [7]. FNH lesions can be hypervascular with portal/delayed washout on imaging, as was seen in our case. As Choi et al. demonstrated [8], 3 out of 9 cases in their series would have been misdiagnosed based on the current AASLD guidelines for the noninvasive diagnostic criteria for HCC. Thus, the presence of a hepatic lesion with arterial hypervascularity and/or portal/delayed washout is not necessarily diagnostic of HCC, particularly in patients in which the clinical picture suggests otherwise.

### 3.3. Proposed Pathogenesis of FNH in Liver Allografts

FNH and FNH-like lesions have been associated with vascular diseases including hereditary hemorrhagic telangiectasia, hepatic hemangiomas, and Budd Chiari syndrome [2, 9, 10]. It has been described in the pediatric population following chemotherapy, venoocclusive disease, and liver radiotherapy [11]. There have also been rare case reports of development following cardiac transplant [12], but few reported cases of FNH-like lesions in posttransplant livers exist. Ra et al. [13] reviewed 4 cases of FNH in posttransplant livers. The time from transplant to FNH diagnosis ranged from 15 to 188 months. Almost all the patients had conditions associated with altered vascular perfusion of the liver, including two with portal vein thromboses and one with a living donor. The authors postulate that the process of transplantation involves significant alterations in vascular perfusion, which could perhaps drive the formation of FNH.

Thus far, research has not shown a direct link between portal vein thromboses and the pathogenesis of FNH. However, evidence suggests that the disruption of liver perfusion causes hepatocyte release of platelet-derived growth factors that can drive hyperplasia. Wanless et al. [14–16] and Kumagai et al. [17] concluded over several studies that FNH pathogenesis begins with a thrombotic insult involving the hepatic artery and/or portal vein leading to regional hepatic ischemia/necrosis. Hepatic arterial recanalization results in transient tissue hyperperfusion. Liver tissues regenerated in these regions with perfusion disturbances all demonstrated a nodular hepatocyte growth pattern suggestive of early FNH formation.

Our patient had an extensive and well-documented history of hypercoagulability (recurrent portal, mesenteric, and splenic vein thromboses, as well as multiple episodes of small bowel infarction requiring surgical resection) dating back at least four years prior to LT. Moreover, recurrence of PVT after transplantation and further episodes of bowel ischemia despite therapeutic anticoagulation suggests continued vascular insults to the liver allograft. We hypothesize that a hyperplastic hepatocyte response ensued, eventually forming an FNH-like lesion.

## 4. Conclusion

We propose that FNH and FNH-like lesions are important considerations in the differential diagnosis of hepatic nodules seen in posttransplant livers, especially in patients with a history of hepatic vascular perfusion compromise. It is incredibly important to consider liver biopsy in patients where the clinical picture does not match the radiologic diagnosis in order to avoid misdiagnosis. The clinical implications of differentiating the lesions from HCC cannot be overstated, especially given the increased morbidity of HCC treatment in the posttransplant setting.
